# Changes in Learning From Social Feedback After Web-Based Interpretation Bias Modification: Secondary Analysis of a Digital Mental Health Intervention Among Individuals With High Social Anxiety Symptoms

**DOI:** 10.2196/44888

**Published:** 2023-08-09

**Authors:** Miranda L Beltzer, Katharine E Daniel, Alexander R Daros, Bethany A Teachman

**Affiliations:** 1 Center for Behavioral Intervention Technologies Northwestern University Feinberg School of Medicine Chicago, IL United States; 2 Department of Psychology University of Virginia Charlottesville, VA United States

**Keywords:** social anxiety, reinforcement learning, cognitive bias modification, interpretation bias, reward learning, probabilistic learning, Q-learning, digital intervention

## Abstract

**Background:**

Biases in social reinforcement learning, or the process of learning to predict and optimize behavior based on rewards and punishments in the social environment, may underlie and maintain some negative cognitive biases that are characteristic of social anxiety. However, little is known about how cognitive and behavioral interventions may change social reinforcement learning in individuals who are anxious.

**Objective:**

This study assessed whether a scalable, web-based cognitive bias modification for interpretations (CBM-I) intervention changed social reinforcement learning biases in participants with high social anxiety symptoms. This study focused on 2 types of social reinforcement learning relevant to social anxiety: learning about other people and learning about one’s own social performance.

**Methods:**

Participants (N=106) completed 2 laboratory sessions, separated by 5 weeks of ecological momentary assessment tracking emotion regulation strategy use and affect. Approximately half (n=51, 48.1%) of the participants completed up to 6 brief daily sessions of CBM-I in week 3. Participants completed a task that assessed social reinforcement learning about other people in both laboratory sessions and a task that assessed social reinforcement learning about one’s own social performance in the second session. Behavioral data from these tasks were computationally modeled using Q-learning and analyzed using mixed effects models.

**Results:**

After the CBM-I intervention, participants updated their beliefs about others more slowly (*P*=.04; Cohen *d*=−0.29) but used what they learned to make more accurate decisions (*P*=.005; Cohen *d*=0.20), choosing rewarding faces more frequently. These effects were not observed among participants who did not complete the CBM-I intervention. Participants who completed the CBM-I intervention also showed less-biased updating about their social performance than participants who did not complete the CBM-I intervention, learning similarly from positive and negative feedback and from feedback on items related to poor versus good social performance. Regardless of the intervention condition, participants at session 2 versus session 1 updated their expectancies about others more from rewarding (*P*=.003; Cohen *d*=0.43) and less from punishing outcomes (*P*=.001; Cohen *d*=−0.47), and they became more accurate at learning to avoid punishing faces (*P*=.001; Cohen *d*=0.20).

**Conclusions:**

Taken together, our results provide initial evidence that there may be some beneficial effects of both the CBM-I intervention and self-tracking of emotion regulation on social reinforcement learning in individuals who are socially anxious, although replication will be important.

## Introduction

### Background

Individuals who are socially anxious tend to see the world through rejection-colored glasses, perceiving ambiguous social situations as more negative than they are and expecting to perform poorly in social situations [1,2]. Some of these negative cognitive biases might be partly maintained by negative learning biases, wherein individuals who are socially anxious have difficulty using positive information to update their previously held negative beliefs [3]. For example, if a person who is socially anxious updated their beliefs about an acquaintance more from the few times that the acquaintance seemed annoyed with them than from the many times the acquaintance was friendly, they might come to have an overly negative expectancy of interactions with that acquaintance. Similarly, negative expectancies about social performance might be partially explained by overweighting instances of negative versus positive feedback—learning more from criticism than praise. Changing these learning processes to be less negatively biased may have positive effects for individuals who are socially anxious, such as changing rigidly negative beliefs about social interactions and performance to be more balanced and reducing motivation to avoid social situations. In this study, we investigated whether a computerized intervention that trains more positive, flexible interpretation styles for individuals who are socially anxious also modifies the learning process by which they update their social expectancies from new experiences (ie, social reinforcement learning [RL]). We used computational modeling to understand changes in particular parameters of interest, specifically learning rates, which are the weights given to positive and negative feedback when updating social expectancies.

### Social RL Biases

RL is the process by which people or other agents learn to predict outcomes and optimize behavior in an environment where taking actions leads to *rewards* (positive outcomes) and *punishments* (negative outcomes) [4]. RL has been used to understand various mental illnesses, including addiction [5], depression [6], and psychosis [7], and has been used to develop algorithms for diverse applications, such as self-driving cars [8], health care decisions [9], and gaming [10]. Social RL is the same learning process but specific to the social environment: updating beliefs about different social actions based on social rewards (such as acceptance and smiles from others) and social punishments (such as rejection and scowls). Previous research has begun to explore social RL in social anxiety, finding that, unlike healthy individuals, who update their beliefs about their social performance more from positive versus negative feedback provided by others, individuals who are socially anxious update more from negative feedback [11]. When confronted with others who evaluate them negatively or neutrally, people who are socially anxious readily learn these evaluations accurately, whereas healthy controls are biased toward believing that others think more positively of them even when they are receiving more negative or neutral evaluations [12]. Some research has found that individuals who are socially anxious tend to learn more from angry versus happy faces [13], whereas other research has not found evidence that people who are socially anxious have biased updating of angry faces [14]. Importantly, several studies have found that individuals who are socially anxious have difficulty adapting their learning and behavior to changing probabilities of social punishment [15-17]. Specifically, there is accumulating evidence that individuals who are socially anxious have difficulty updating negative social expectancies to become more positive [14,18]. Together, these studies suggest that individuals who are socially anxious may have negative biases in social RL that can make it difficult to learn from social interactions, even when they seemingly go well.

Although there is considerable literature on the neural underpinnings of RL and how pharmacological interventions may change RL, less is known about changing RL through cognitive and behavioral interventions. Several pharmacological interventions have been used to normalize aberrant processing of rewards and punishments in various psychological disorders [19-22], including specifically changing punishment learning rates (ie, shifting how heavily a person weighs a negative outcome when updating expectancies) [23,24]. However, very little research has assessed whether cognitive behavioral interventions change RL processes, despite several researchers advocating for computationally modeling learning and cognitive change in cognitive behavioral therapy [25,26]. Recent studies have shown that cognitive interventions can change RL processes in healthy individuals [27] and individuals with schizophrenia [28]. To our knowledge, this study is the first to behaviorally assess changes in RL through cognitive bias modification in a population with elevated anxiety symptoms.

### Cognitive Bias Modification

This study assesses whether several social RL biases, which are biases in learning from positive and negative social feedback to update beliefs about others and one’s own social performance, can be changed through a scalable, computerized intervention called cognitive bias modification for interpretations (CBM-I). CBM-I programs aim to train people to make less threatening and more flexible interpretations of ambiguous stimuli. They often do this by presenting scenarios that are emotionally ambiguous until the final word. The final word more frequently resolves the ambiguity in positive or neutral ways than negative ways, which aims to train participants to interpret ambiguous situations more benignly. CBM-I is a targeted, mechanism-driven intervention that is easily scalable. Because individuals who are socially anxious can use this intervention from the comfort of their own homes or wherever it is convenient for them, CBM-I holds potential either as an adjunct to other treatments or as a stand-alone intervention. CBM-I can effectively reduce cognitive biases and, to a lesser extent, the symptoms of social anxiety disorder [29-32]. However, there has been mixed evidence regarding the efficacy of CBM-I, including small effect sizes [33,34].

CBM-I is a useful intervention to study changes in social RL because the cognitive mechanism is conceptually related. Training people to interpret ambiguous situations more benignly may facilitate changes in social RL by priming awareness of potential social rewards and reducing expectations of social rejection. For example, in the CBM-I intervention used in this study, participants read emotionally ambiguous scenarios in which the ambiguity is only resolved in the final word, which is missing a letter that participants complete (eg, “You are required to give a presentation to a group of work colleagues that you know well. They all are quiet during your presentation. As you think about the presentation later at home, you think that your colleagues found your presentation...stimul_ting”). As a participant who is socially anxious reads this scenario, they might start to think the scenario will end poorly because they expect punishment in socially threatening situations (eg, that their colleagues hated their presentation), but the actual resolution of the ambiguity in a rewarding way (ie, that their colleagues found their presentation stimulating) might train the participant to update their future expectancies to be less threatening. Notably, previous work has found that RL parameters predict the response to cognitive bias modification to shift attention bias (ie, selective focus on threat cues) [35]; given their similar focus on shifting threat biases, CBM-I may also be related to RL.

### Evidence for Social RL and CBM-I Change in These Data

Three previous studies [36,37] (Beltzer, ML, unpublished data, June 2023) that are relevant to this study have been performed using data from the same data set, and these studies informed our hypotheses. Two of these studies [36] (Beltzer, ML, unpublished data, June 2023) assessed social anxiety–related differences in social RL by comparing participants with high social anxiety symptoms (who had not completed CBM-I) with participants with low social anxiety symptoms on 2 social RL tasks. First, the social probabilistic selection task assessed learning about other people by asking participants to choose between neutral faces with different probabilities of becoming happy or angry when chosen. Second, the speech expectancies task assessed learning about one’s own social performance by asking participants to rate how they expected to perform on a stressful speech both before the speech and again after the speech and seeing feedback from the judges on their performance (the tasks are fully described in the *Methods* section and in their original papers [36] [Beltzer, ML, unpublished data, June 2023]). For each of these tasks, we used RL to computationally model how each participant weighted rewarding and punishing social outcomes when updating their beliefs, parameterized as the reward and punishment learning rates. These studies did not find social anxiety–related differences in learning rates as a function of social anxiety group or whether social feedback was rewarding or punishing but did find that, contrary to hypotheses, participants with high versus low social anxiety symptoms were less accurate at avoiding punishing faces once feedback was no longer given [36]. Exploratory analyses found that participants (regardless of social anxiety group) updated their expectancies of their social performance more from positive than negative feedback and that participants with high versus low social anxiety symptoms used feedback to update their expectancies more for elements of poor social performance than good social performance (Beltzer, ML, unpublished data, June 2023). These results suggest that negative social RL biases were not evident in this sample in the ways we had expected; instead, participants with high social anxiety showed impaired punishment learning accuracy and greater updating from feedback about feared, negative aspects of social performance.

Another study from this data set [37] assessed the effects of CBM-I on outcomes related to cognitive styles and social anxiety symptoms. Participants who completed both CBM-I and ecological momentary assessment (EMA) about their emotion regulation experienced greater reductions in trait negative interpretation bias than participants who completed only EMA. However, CBM-I was not uniquely related to changes in other trait cognitive styles (ie, cognitive flexibility, reappraisal ability, social interaction anxiety, and fear of negative evaluation). CBM-I also increased the daily self-reported ability to use cognitive reappraisal but it did not change other daily cognitive style outcomes more than only EMA. These results suggest that, in this data set, the CBM-I intervention effectively changed negative interpretation biases but did not have effects on many other processes related to social anxiety that it did not target as directly. Given social RL’s close conceptual ties to interpretation biases, it is possible that although null effects were found on many of these peripheral outcomes, CBM-I might still exert effects on social RL.

### Overview and Hypotheses

This study examined whether social RL biases changed as a function of completing a week of CBM-I in the middle of a 5-week EMA study tracking emotion regulation strategy use and affect (as compared with the comparison condition that also completed 5 weeks of EMA but no CBM-I). We assessed whether CBM-I was associated with differences in the weights given to socially rewarding versus punishing information when updating beliefs in 2 domains: learning about other people and learning about one’s own social performance. Given the slight methodological differences, these are referred to as “learning rates” for learning about other people and “update weights” for updating social performance expectancies based on feedback. In the domain of learning about other people, we also assessed whether CBM-I was associated with differences in using what one has learned about others to guide one’s own behavior to accurately choose rewarding people and to avoid punishing people. To do this, we compared parameters extracted from a social probabilistic learning task administered at baseline and follow-up laboratory sessions, and a speech expectancy updating task administered at follow-up, as a function of the intervention group (CBM-I vs only EMA).

To our knowledge, no study to date has assessed changes in RL as a function of CBM-I. We had competing hypotheses for most outcomes, as prior analyses of social RL in this data set found results that diverged from hypotheses based on extant literature. Plans for analyses and competing hypotheses were preregistered on the web [38].

On the basis of conceptually related findings of decreased negative interpretation bias after completing a course of CBM-I (observed both in the literature and in this data set, as described earlier) [37], we hypothesized that participants who are socially anxious would exhibit a decrease in parameters related to social punishment learning (punishment learning rate and accuracy in avoiding punishment) and/or an increase in parameters related to social reward learning (reward learning rate and accuracy in choosing reward). Modeling these parameters separately allowed us to pinpoint whether CBM-I led to changes in learning from social reward and/or punishment and whether those changes were reflected in the weight given to new social information and/or the accuracy of decisions made based on information learned about reward and/or punishment. Similarly, we hypothesized that socially anxious participants who completed CBM-I would update their beliefs about their own social performance more based on positive feedback and/or less based on negative feedback, as compared with the EMA-only group.

On the basis of our prior analyses on the social probabilistic learning task, finding no differences in learning rates as a function of the social anxiety group, we hypothesized that we would not find a difference between the intervention groups in learning rates at either session. On the basis of our surprising finding of impaired accuracy in avoiding punishment in the high social anxiety group [36], we hypothesized that CBM-I might mitigate this bias, evidenced as a greater increase in accuracy in avoiding punishment from baseline to follow-up in the CBM-I versus EMA-only group. On the basis of our prior exploratory finding of greater updating on items assessing poor social performance in the high versus low social anxiety group (Beltzer, ML, unpublished data, June 2023), we hypothesized that a model with 2 update weights (estimated separately for items assessing good vs poor social performance) would reveal a CBM-I versus EMA-only intervention–related difference. Specifically, we hypothesized that CBM-I would mitigate this bias toward greater updating on items assessing poor social performance, such that the CBM-I versus EMA-only group would update their expectancies less from feedback on items measuring poor versus good performance.

## Methods

### Participants

#### Overview

Participants were recruited through the University of Virginia undergraduate participant pool, advertisements sent to university email lists for undergraduate and graduate students, and flyers posted in public areas around the community. Prospective participants completed a screening survey, and 114 adults (aged 18-45 years) with moderate to severe social anxiety symptoms were invited to participate based on eligibility criteria defined a priori (scoring 29 or greater out of a possible 80 points on the Social Interaction Anxiety Scale, approximately one-fourth of an SD below the mean in a sample diagnosed with social phobia [39]; see Table 1 for demographic information about the sample and Multimedia Appendix 1 for the CONSORT [Consolidated Standards of Reporting Trials] diagram and details about dropout).

**Table 1:** Demographic characteristics of the final sample of 106 participants analyzed.

		CBM-I^a^ (n=51)	Only EMA^b^ (n=55)
**Sex, n (%)**			
	Female	38 (74.51)	40 (72.73)
	Male	13 (25.49)	15 (27.27)
	Nonbinary	0 (0)	0 (0)
Age (years), mean (SD)		20.67 (2.92)	20.24 (3.12)
Undergraduates, n (%)		36 (70.59)	46 (83.64)
**Ethnicity, n (%)**			
	Latinx or Hispanic	1 (1.96)	2 (3.64)
	Not Latinx or Hispanic	50 (98.04)	52 (92.55)
	Prefer not to answer	0 (0)	1 (3.81)
**Race, n (%)**			
	African American or Black	2 (3.92)	6 (10.91)
	Asian	11 (21.57)	10 (18.18)
	Hawaiian or Pacific Islander	1 (1.96)	2 (3.64)
	Middle Eastern	1 (1.96)	2 (3.64)
	Native American	0 (0)	0 (0)
	White	39 (76.47)	40 (72.73)

^a^CBM-I: cognitive bias modification for interpretations.

^b^EMA: ecological momentary assessment.

### Procedure

All participants completed a baseline session in the laboratory that included informed consent, questionnaires, and social probabilistic selection and speech expectancy tasks. Participants then completed 5 weeks of EMA about their emotion regulation (for more information on EMA procedures, see the studies by Daniel et al [37], Beltzer et al [40], and Daniel et al [41]), followed by another laboratory session with the same tasks (because the mobile app used for only EMA ran on certain versions of iOS and Android OS, participants who did not have a compatible smartphone were excluded. More information about the EMA component of the study, as well as other measures administered for the larger parent study but not analyzed in this study, can be found on the web [42]). Approximately half (59/114, 51.8%) of the sample was randomized to complete a week-long CBM-I intervention during week 3, and this CBM-I condition’s EMA prompts were slightly reduced during this week to ensure that the time spent on tasks was similar across conditions.

#### Cognitive Bias Modification

Over the course of week 3, participants assigned to the CBM-I condition were asked to complete six 10- to 15-minute web-based CBM-I sessions on their PC or smartphone, once per day. Participants read a series of 30 ambiguous scenarios that raised the possibility of a threat (18 social, 7 physical, and 5 other threat), which only became disambiguated once the final word of the scenario was read, from which 1 or 2 letters were missing (eg, “While at the hairdresser’s, you opt for a completely different haircut. When you see your friend afterwards, she gasps. Her gasp probably means that she thinks the new style makes you look...gre_t”). After filling in the correct missing letter or letters to resolve the scenario’s emotional ambiguity (eg, “gre*a*t”), the participant was asked a yes-or-no or multiple-choice question about the scenario to ensure the participant had read it and understood the disambiguated ending. The disambiguation resolved the ambiguity in a benign, nonthreatening way for 90% of scenarios, allowing participants to learn through practice that uncertain situations can end in a variety of ways (including in more rewarding ways than expected, counteracting their biased negative expectations). The full list of CBM-I materials (including scenarios, disambiguating words, and comprehension questions) is available on the web [43]. See Multimedia Appendix 2 for screenshots of the CBM-I training.

### Measures

#### Social Probabilistic Selection Task

The social probabilistic selection task [13] was used to assess social RL about other people. This was an adaptation of a widely used probabilistic category learning paradigm [44] that uses socially relevant information as stimuli (neutral faces) and socially evaluative reinforcement as feedback (reward: happy faces, punishment: angry faces). The task consisted of 2 phases: training and testing. In the training phase, participants were presented with 2 neutral faces at a time and were instructed that one face would become happy if chosen but the other would become angry. They were instructed to select the face that they thought was more likely to be happy. The pairs of faces had different complementary reward contingencies (ie, in 1 pair, one face became happy 80% of the time it was chosen and angry 20% and the other face became happy 20% and angry 80%; the other pairs were similar but with 70%/30% and 60%/40% contingencies) but participants had to learn which faces to choose through trial and error rather than explicitly being told the contingencies. In the testing phase, the faces were recombined into all possible pairs (eg, the 80% rewarding face was presented in pairs with 70%, 60%, 40%, 30%, and 20% rewarding faces, rather than just the 20% rewarding face, as in the training phase). Participants were instructed to select the more rewarding face based on what they had learned during the training phase, and no feedback was given (ie, the neutral faces did not become happy or angry). See Multimedia Appendix 3 [11,13,44-49] for more details on the social probabilistic selection task.

#### Speech Expectancies Task

The speech expectancies task (modified from Koban et al [11]) was used to assess social RL about one’s own social performance. Modified from the Trier Social Stress Test [50], participants had 2 minutes to mentally prepare to give a stressful, 4-minute speech for a panel of 2 confederate judges, who video recorded the speech and were instructed to maintain neutral facial expressions throughout. Before the speech, participants rated how they expected to perform on 10 items related to good social performance (eg, “I will appear calm”) and 10 related to poor performance (eg, “I will appear to be sweating”) on a scale from 0 (“disagree”) to 100 (“agree”; adapted from Cody and Teachman [51]).

After the speech at the second laboratory session, participants received false feedback on their performance, which was ostensibly from the judges but was actually randomly generated to fall within a range around the participant’s prespeech self-ratings to be believable [11]. For each social performance–related item, participants were asked to rate how they expected to perform on a similar speech in the future. Before entering their postspeech expectancy rating, they were first shown their prespeech rating and then the false feedback. To generate the false feedback, a random integer between −50 and 50 was added to each participant’s self-rating on each item, bounded by the 0 to 100 rating scale, to obtain the feedback rating. Speech ratings and feedback were presented in PsychoPy2 [52]. Participants only completed the false feedback and postspeech expectancy ratings in session 2 because we felt it was not ethical to use deception in session 1 and then not debrief participants until 5 weeks later at the end of the study. See Multimedia Appendix 3 for more details about the speech expectancies task.

### Ethics Approval

This study was approved by the University of Virginia Institutional Review Board (UVA IRB 2270). and was conducted in accordance with the ethical standards of the responsible committee on human experimentation (institutional and national) and the Helsinki Declaration of 1975, as revised in 2000.

### Informed Consent and Participation

All the participants provided informed consent. Participants were compensated US $25 for each laboratory session they completed and up to US $70 for completing EMA surveys and CBM-I sessions, with more compensation for higher completion, as detailed in the informed consent form, for a total compensation of up to US $120 for the completion of all study components. Participants who were undergraduate students at University of Virginia could alternatively claim up to 4 hours of research credits for participation. Data were deidentified for analysis. Patients were fully debriefed at the end of the study. As described earlier, the feedback and expectancy updating portion of the speech expectancies task was administered only at each participant’s final session to be able to debrief about false feedback on the same day they received feedback.

### Plan for Analyses

#### Overview

This study assessed whether individuals who are socially anxious who completed CBM-I plus EMA versus only EMA learned differently from positive and negative social feedback when updating their expectancies about other people and their own social performance. To do this, we applied versions of a Q-learning model to the social probabilistic selection task and the speech expectancies task. We then compared parameters related to learning (ie, learning rates and update weights for positive and negative feedback) between groups. In the domain of learning about other people, we also assessed whether CBM-I changed the participants’ accuracy in choosing socially rewarding and avoiding socially punishing faces during the testing phase of the social probabilistic selection task. Reward and punishment learning accuracy were defined as the proportion of times the participant accurately selected the more rewarding face when the most rewarding (80% happy) and most punishing (80% angry) faces, respectively, were paired with all intermediate faces (70%, 60%, 40%, and 30%) during the testing phase.

#### Computational Modeling of the Social Probabilistic Selection Task

The social probabilistic selection task was computationally modeled using the *hBayesDM* R package [53], which includes hierarchical Bayesian modeling of the Q-learning estimation procedure [54]. Q-learning models the process of updating the expected values of each neutral face by learning from each experience of a rewarding (eg, happy) or punishing (eg, angry) outcome. We compared 2 candidate models. One model fitted the participants’ choice data from the training phase of the task with separate reward and punishment learning rate parameters, which, respectively, represent the weights given to positive and negative prediction errors (the value difference between the observed and expected outcomes) when updating these expected values. The other model included a single learning rate parameter for all trials (and so was nested in the more complex model). We compared the prediction accuracy for each condition at each session using the leave-one-out information criterion (LOOIC), given our relatively small sample size [55]. Because the more complex model had a lower LOOIC for one group and the models provided a relatively similar fit for the other groups, the more complex model was chosen (see Multimedia Appendix 3 for LOOIC values and for more detail about computational modeling of this task).

#### Computational Modeling of the Speech Expectancies Task

For the speech expectancies task, we used similar models to estimate how participants learned from feedback when updating their postspeech expectancies of how they would perform on a future speech. Given concerns about having too few items per cell to reliably model separate update weights for positive and negative feedback, we modeled update weights in 2 ways: separately for feedback that was more positive than participants’ prespeech self-ratings (ie, positive prediction errors) and feedback that was more negative than participants’ prespeech self-ratings (ie, negative prediction errors) and separately for items measuring good versus poor speech performance (to be thorough, we also ran models with 1 update weight estimated across all 20 items and with 4 update weights estimated [one for each combination of prediction error and item valence]; see Multimedia Appendix 3 for more details). For items measuring good speech performance, a positive prediction error occurred when the feedback rating was higher than the participant’s prespeech self-rating; for items measuring poor speech performance, this was reversed, so a positive prediction error occurred when the feedback rating was lower than the participant’s prespeech self-rating. A similar classification procedure was used to identify negative prediction errors, with the scoring reversed for items measuring poor speech performance. Note that the update weights were similar to the learning rates modeled for the social probabilistic selection task but were estimated over only 1 instance of updating per item. See the Multimedia Appendix 3 for more details on the computational modeling of this task.

## Results

### Analyses of the Social Probabilistic Selection Task (RL About Other People)

Participants’ behavior on the social probabilistic selection task was analyzed to better understand how CBM-I affected social RL about other people. Specifically, we assessed reward and punishment learning rates, which described the weights given to happy and angry faces, respectively, in updating expectancies of other people during the training phase and accuracy in choosing rewarding and avoiding punishing faces in the testing phase.

#### Learning Rates Estimated Over the Training Phase

**Figure 1:**
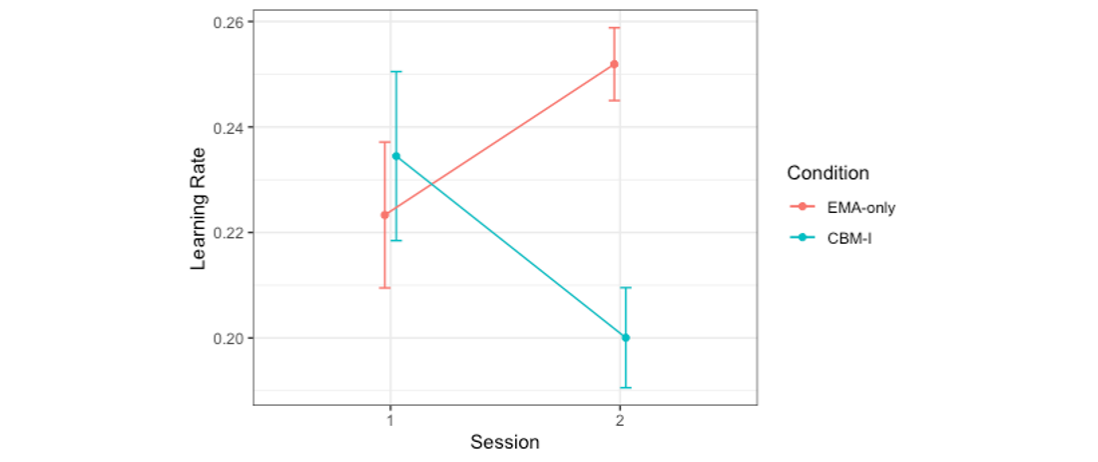
Learning rates in the social probabilistic selection task decreased from session 1 to session 2 in the cognitive bias modification for interpretations (CBM-I) condition but did not significantly change in the ecological momentary assessment (EMA)–only condition. Condition=intervention condition (CBM-I vs only EMA).

**Figure 2:**
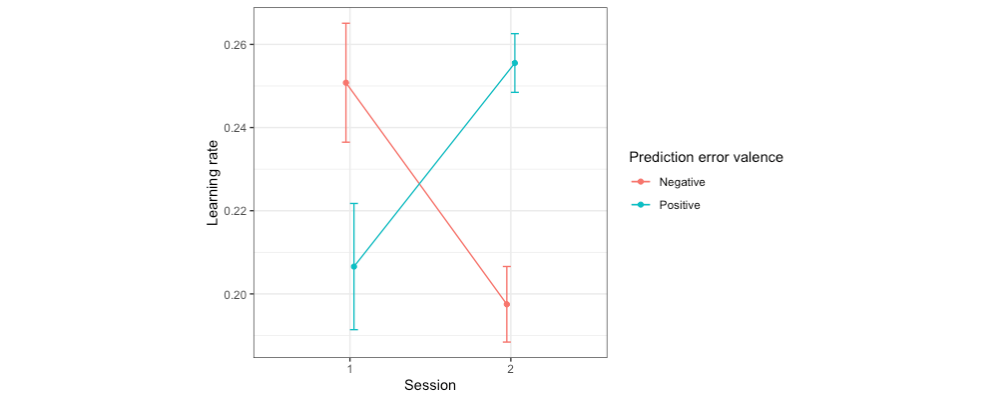
Punishment learning rates decreased from session 1 to session 2, whereas reward learning rates increased from session 1 to session 2 in the social probabilistic selection task. CBM-I: cognitive bias modification for interpretations; EMA: ecological momentary assessment.

To assess learning rate differences as a function of CBM-I condition, a mixed effects model was used to predict learning rate from fixed effects of CBM-I condition, session, prediction error valence, and all 2- and 3-way interactions, with a random intercept for participant. There were significant session-by-condition and session-by-prediction error valence interactions. Post hoc pairwise comparisons of estimated marginal means (with a Tukey adjustment; for all post hoc tests, note that estimated marginal means and odds ratios are presented on the logit scale) revealed that, in the CBM-I condition, learning rates decreased from session 1 (mean 0.23, SE 0.01) to session 2 (mean 0.20, SE 0.01; t_303_=−2.04; *P*=.04; Cohen *d*=−0.29) but did not significantly change in the EMA-only condition (session 1: mean 0.22, SE 0.01; session 2: mean 0.25, SE 0.01; t_308_=1.79; *P*=.07; Cohen *d*=0.25; Figure 1). In other words, after completing CBM-I, participants updated their expectancies about other people less based on feedback, regardless of whether that feedback was a happy or angry face. Punishment learning rates decreased from session 1 (mean 0.25, SE 0.01) to session 2 (mean 0.20, SE 0.01; t_299_=−3.34; *P*=.001; Cohen *d*=−0.47), whereas reward learning rates increased from session 1 (mean 0.21, SE 0.01) to session 2 (mean 0.26, SE 0.01; t_299_=3.04; *P*=.003; Cohen *d*=0.43; Figure 2). This means that by the end of the study, participants (regardless of the intervention condition) updated their expectancies about others less from angry faces and more from happy faces (Table 2). These findings are partially in line with hypotheses based on prior literature. We hypothesized that punishment learning rates would decrease and/or reward learning rates would increase in the CBM-I condition, whereas the results suggested that both of these changes were observed regardless of the intervention condition (rather than being specific to CBM-I). Furthermore, learning rates decreased in the CBM-I condition, regardless of the prediction error valence (rather than being specific to negative prediction errors).

**Table 2:** Model estimates predicting learning rate in the training phase of the social probabilistic selection task (n=106 participants)^a^.

Predictors		Learning rate		
		Estimates (95% CI)	*t* test (*df*)	*P* value
**Fixed effects**				
	Intercept	0.23 (0.21 to 0.24)	32.10 (100.88)	*<.001* ^b^
	Session	−0.00 (−0.01 to 0.01)	−0.21 (305.78)	.83
	Condition^c^	−0.01(−0.02 to 0.00)	−1.39 (100.88)	.16
	PE valence^d^	0.00 (−0.01 to 0.01)	0.63 (291.80)	.53
	Session × condition	−0.02 (−0.03 to −0.00)	−2.71 (305.78)	*.007*
	Session × PE valence	0.03 (0.01 to 0.04)	4.53 (291.80)	*<.001*
	Condition × PE valence	0.01 (−0.00 to 0.02)	1.13 (291.80)	.26
	Session × condition × PE valence	0.01 (−0.00 to 0.02)	1.16 (291.80)	.25

^a^Random effects: σ^2^=0.01, τ_00_ (participant)=0.00, intraclass correlation coefficient=0.13, observations=404, marginal R^2^=0.067, conditional R^2^=0.185.

^b^Italicization indicates *P*<.05.

^c^Condition: intervention condition (cognitive bias modification for interpretations vs only ecological momentary assessment).

^d^PE valence: prediction error valence (positive vs negative).

#### Accuracy Measured During the Testing Phase

To assess differences in accuracy at choosing the most rewarding face and avoiding the most punishing face in the testing phase as a function of the CBM-I condition, a generalized linear mixed model was used to predict the proportion of times the participant accurately chose the most rewarding face and accurately did not choose the most punishing face when each of them was paired with all other faces in the testing phase. This model included fixed effects of the CBM-I condition, session, prediction error valence, and all 2- and 3-way interactions, with a random intercept for participant. There was a significant main effect of trial type (choose reward vs avoid punishment) that was not interpreted because it was subsumed within a significant interaction (Table 3), which more thoroughly and accurately describes the effects [56]. There were statistically significant session-by-condition, session–by–trial type, and condition–by–trial type interactions.

**Table 3:** Model estimates predicting accuracy in the testing phase of the social probabilistic selection task (n=106 participants)^a^.

Predictors		Probability of selecting the more rewarding face		
		Odds ratio (95% CI)	*z* score	*P* value
**Fixed effects**				
	Intercept	4.55 (3.85-5.38)	17.71	*<.001* ^b^
	Session	1.04 (0.99-1.09)	1.67	.09
	Condition^c^	1.05 (0.89-1.25)	0.62	.54
	Trial type^d^	1.33 (1.27-1.39)	12.25	*<.001*
	Session × condition	1.06 (1.01-1.11)	2.44	*.02*
	Session × trial type	0.94 (0.90-0.98)	−2.67	*.008*
	Condition × trial type	1.09 (1.04-1.14)	3.54	*<.001*
	Session × condition × trial type	1.02 (0.98-1.07)	0.99	.32

^a^Random effects: σ^2^=3.29, τ_00_ (participant)=0.70, intraclass correlation coefficient=0.18, observations=12,863, marginal *R*^2^=0.024, conditional *R*^2^=0.195.

^b^Italicization indicates *P*<.05.

^c^Condition: intervention condition (cognitive bias modification for interpretations vs only ecological momentary assessment).

^d^Trial type: choose reward versus avoid punishment.

**Figure 3:**
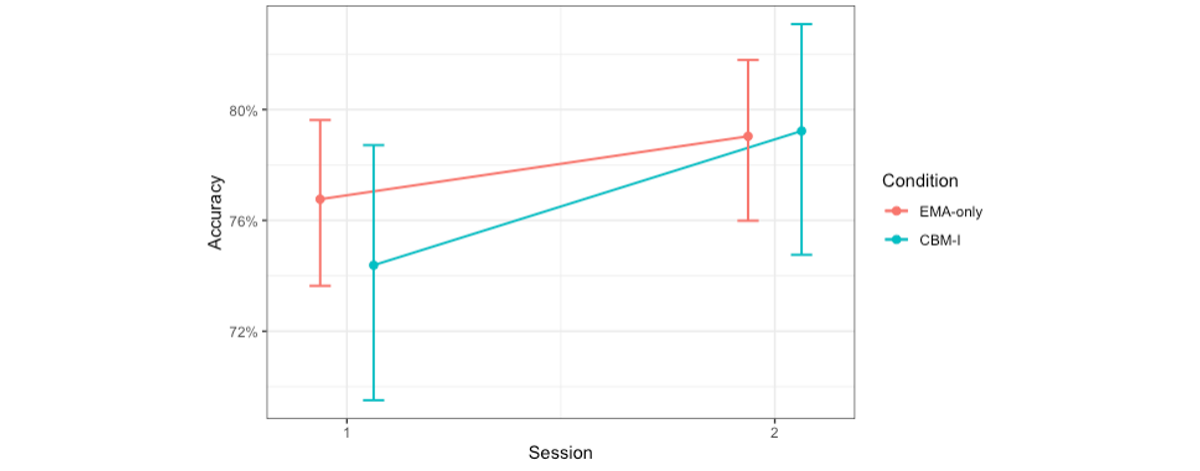
Decision accuracy in the testing phase of the probabilistic selection task (percentage of times the more rewarding face was chosen from pairs that included intermediate faces paired with either the most rewarding face or the most punishing face) increased from session 1 to session 2 in the cognitive bias modification for interpretations (CBM-I) condition, but did not significantly change in the ecological momentary assessment (EMA)–only condition.

To better understand these statistically significant interactions, we performed post hoc pairwise comparisons of the estimated marginal means (with a Tukey adjustment). We report those relevant to our hypotheses (those that include session) here and the less-relevant interactions in Multimedia Appendix 3. Following up on the session-by-condition interaction, we found that testing phase accuracy increased from session 1 (mean 1.47, SD 0.13) to session 2 (mean 1.67, SD 0.13; z score 2.84; *P*=.005; Cohen *d*=0.20) in the CBM-I condition but did not change in the EMA-only condition (session 1: mean 1.48, SD 0.12; session 2: mean 1.44, SD 0.12; z score 0.56; *P*=.58; Cohen *d*=−0.04; Figure 3). In other words, after completing CBM-I, participants used the probabilities they had learned to make better decisions about other people in the testing phase, regardless of whether that meant choosing rewarding faces or avoiding punishing faces.

**Figure 4:**
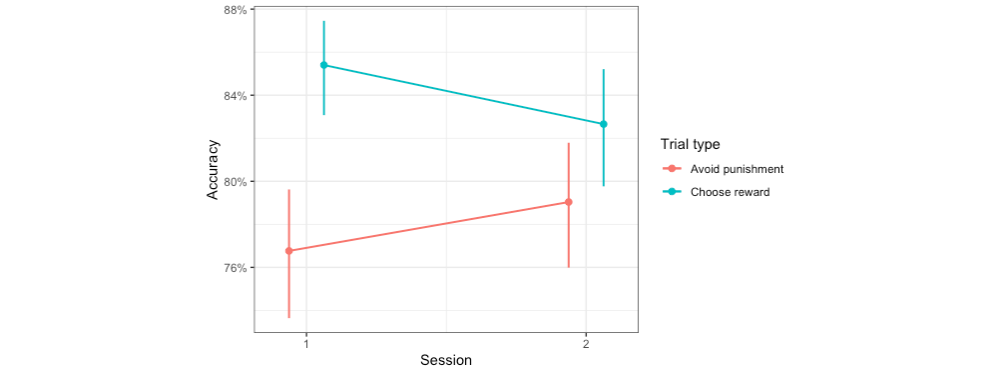
Accuracy in avoiding the most punishing face in the testing phase of the probabilistic selection task increased from session 1 to session 2, but accuracy in choosing the most rewarding face did not significantly change across sessions.

Following up on the session–by–trial type interaction, we found that accuracy in avoiding punishment increased from session 1 (mean 1.13, SD 0.09) to session 2 (mean 1.33, *SD* 0.09; *z* score 3.28; *P*=.001; Cohen *d*=0.20) but accuracy in choosing rewards did not (session 1: mean 1.82, SD 0.10; session 2: mean 1.78, SD 0.10; z score −0.62; *P*=.54; Cohen *d*=−0.04; Figure 4). This suggests that by the end of the study, participants (regardless of the CBM-I condition) became better at learning to avoid the most punishing face. These results are partially in line with the hypotheses based on prior studies using this data set. We hypothesized that accuracy in avoiding punishment would increase for the CBM-I condition, whereas the results suggested that accuracy (not specific to punishment) increased for the CBM-I condition, and accuracy in avoiding punishment increased but not specifically for the CBM-I condition.

### Analyses of the Speech Expectancies Task (RL About One’s Own Social Performance)

#### Overview

Self-ratings before and after feedback on the speech expectancies task were analyzed to better understand how CBM-I affected social RL about one’s own social performance. Specifically, we assessed update weights describing the extent to which feedback from the judges was weighted when participants updated their expectancies of their own public-speaking performance. These update weights were conceptually similar to the learning rates on the social probabilistic selection task but described learning in a different domain (about one’s own social performance, rather than about other people). Because feedback was only given during the speech expectancies task in session 2, we did not have a session-1 measure of social RL about one’s own social performance. However, we were still able to analyze the differences at session 2 between the CBM-I and EMA-only groups.

#### Update Weights Estimated Separately by Prediction Error Valence

**Figure 5:**
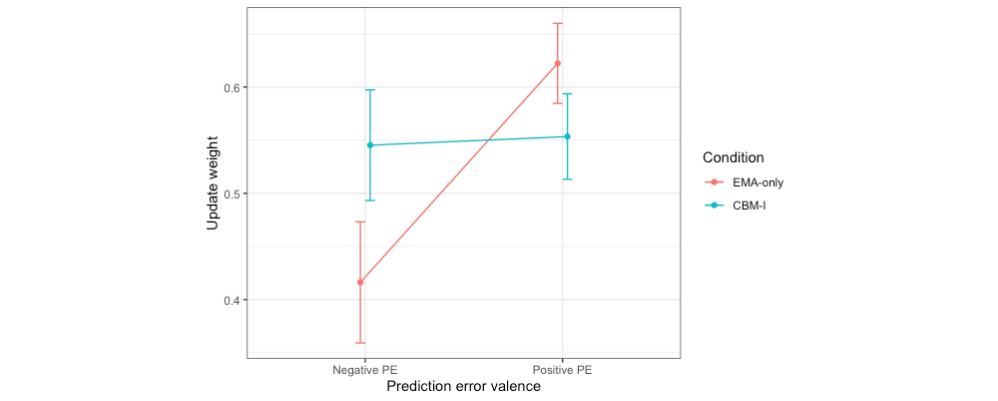
Update weights in the 2 weights (prediction error valence) model for the speech expectancies task. Condition=intervention condition (cognitive bias modification for interpretations [CBM-I] vs only ecological momentary assessment [EMA]). In the 2 weights (prediction error valence) model, update weights were estimated separately for items on which feedback was more negative versus positive than participants’ prespeech expectancies (prediction error valence), without regard for item valence (items measuring good vs poor social performance). PE: prediction error.

For update weights estimated separately by prediction error valence, a mixed effects model was used to predict update weights from fixed effects of the CBM-I condition, prediction error valence, and their interaction, with a random intercept for participant. Nine influential outliers were removed. A significant main effect of prediction error valence was observed but was not interpreted because it was subsumed within a significant interaction with the CBM-I condition. Post hoc pairwise comparisons of the estimated marginal means (with a Tukey adjustment) revealed that, contrary to hypotheses based on prior literature, participants in the EMA-only condition updated their expectancies about their social performance more from positive (mean 0.62, SE 0.05) than negative feedback (mean 0.42, SE 0.05; t_68.9_=3.43; *P*=.001; Cohen *d*=−0.78), whereas participants in the CBM-I condition updated similarly from positive (mean 0.55, SE 0.05) and negative feedback (mean 0.54, SE 0.05; t_68.4_=0.20; *P*=.85; Cohen *d*=−0.05; Figure 5). This suggests that EMA-only participants showed a bias toward learning from positive feedback about their social performance, whereas CBM-I participants did not learn significantly differently from positive versus negative feedback (Table 4).

**Table 4:** Model estimates predicting update weights estimated separately by prediction error valence in the speech expectancies task (n=78 participants)^a^.

Predictors		Update weight		
		Estimates (95% CI)	*t* test (*df*)	*P* value
**Fixed effects**				
	Intercept	0.53 (0.48 to 0.58)	20.15 (70.93)	*<.001* ^b^
	PE valence	0.05 (0.01 to 0.09)	2.48 (68.64)	*.01*
	Condition^c^	0.01 (−0.04 to 0.07)	0.56 (70.93)	.58
	PE valence^d^ × condition	−0.05 (−0.09 to −0.00)	−2.19 (68.64)	*.03*

^a^Random effects: σ^2^=0.06, τ_00_ (participant)=0.02, intraclass correlation coefficient=0.24, observations=147, marginal *R*^2^=0.061, conditional *R*^2^=0.286.

^b^Italicization indicates *P*<.05.

^c^Condition: intervention condition (cognitive bias modification for interpretations vs only ecological momentary assessment).

^d^PE valence: prediction error valence (positive vs negative).

#### Update Weights Estimated Separately by Item Valence

**Figure 6:**
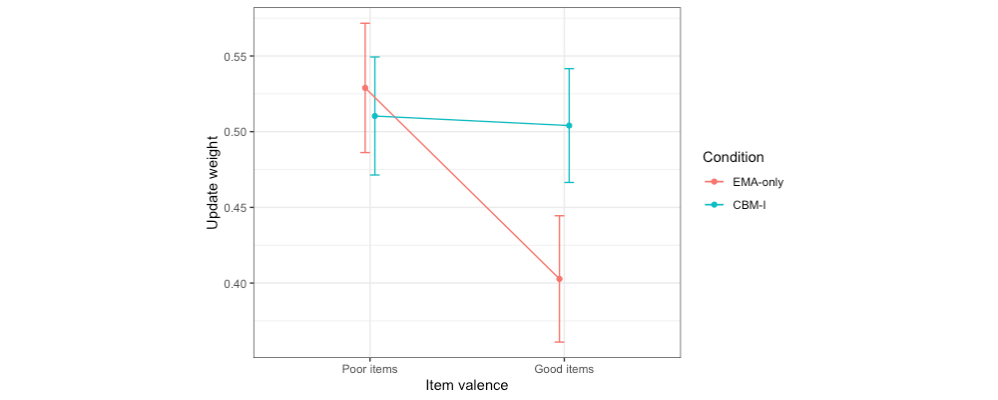
Update weights in the 2 weights (item valence) model for the speech expectancies task. Condition=intervention condition (cognitive bias modification for interpretations [CBM-I] vs only ecological momentary assessment [EMA]). In the 2 weights (item valence) model, update weights were estimated separately for items measuring good versus poor social performance, without regard for prediction error valence (whether feedback was more negative vs positive than participants’ prespeech expectancies).

For update weights estimated separately by item valence, a mixed effects model was used to predict update weights from fixed effects of the CBM-I condition, item valence, and their interaction, with a random intercept for participant. Ten influential outliers were removed. A significant main effect of item valence was observed but was not interpreted because it was subsumed within a significant interaction with the CBM-I condition. Post hoc pairwise comparisons of the estimated marginal means (with a Tukey adjustment) revealed that, in line with the hypotheses, participants in the EMA-only condition updated their expectancies more from feedback on items measuring poor (mean 0.52, SE 0.04) versus good social performance (mean 0.41, SE 0.04; t_70.2_=−4.60; *P*<.001; Cohen *d*=1.04), whereas participants in the CBM-I condition updated similarly from feedback on both types of items (poor items: mean 0.51, SE 0.04; good items: mean 0.51, SE 0.04; t_69.6_=−0.12; *P*=.91; Cohen *d*=0.03; Figure 6). This suggests that EMA-only participants showed a bias toward learning from feedback about poor aspects of social performance, such as speaking too quickly, versus good aspects, such as seeming confident, whereas CBM-I participants did not learn significantly differently from feedback on poor versus good items (Table 5).

**Table 5:** Model estimates predicting update weights estimated separately by item valence in the speech expectancies task (n=75 participants)^a^.

Predictors		Update weight		
		Estimates (95% CI)	*t* test (*df*)	*P* value
**Fixed effects**				
	Intercept	0.49 (0.43 to 0.54)	17.81 (73.06)	*<.001* ^b^
	Item valence	−0.03 (−0.05 to −0.01)	−3.17 (69.83)	*.002*
	Condition^c^	0.02 (−0.03 to 0.08)	0.79 (73.06)	.43
	Item valence × condition^d^	0.03 (0.01 to 0.05)	3.00 (69.83)	*.003*

^a^Random effects: σ^2^=0.01, τ_00_ (participant)=0.05, intraclass correlation coefficient=0.80, observations=146, marginal *R*^2^=0.036, conditional *R*^2^=0.810.

^b^Italicization indicates *P*<.05.

^c^Condition: intervention condition (cognitive bias modification for interpretations vs only ecological momentary assessment).

^d^Item valence: items measuring good versus poor social performance.

## Discussion

### Overview

This study assessed the effects of CBM-I on social RL in individuals who are socially anxious, focusing on 2 important domains: learning about other people and learning about one’s own social performance. For the most part, we did not find evidence supporting the 3-way interactions that we had hypothesized (ie, interactions among session, intervention condition, and prediction error valence), but we did find evidence of several related 2-way interactions. After versus before CBM-I, participants updated their expectancies of other people on the social probabilistic selection task more slowly from new social information and used what they learned to make more accurate decisions. These changes were not observed in the EMA-only condition, suggesting that they were likely related to the CBM-I intervention. Furthermore, participants who completed CBM-I used social feedback to update their expectancies of their own performance on the speech expectancies task in less-biased ways than EMA-only participants, in that they learned similarly from positive and negative feedback and learned similarly from feedback on items related to poor and good social performance. We also observed several effects as a function of session (ie, change over time), regardless of the intervention condition, which might be a result of either practice with the social probabilistic selection task or from the 5 weeks of EMA tracking affect and emotion regulation that all participants completed. Specifically, at the end of the study, participants updated their expectancies about others more from reward and less from punishment, and they became more accurate at avoiding punishment, as compared to at the start of the study. Although the results were mixed, these findings point to several intriguing changes in social RL associated with CBM-I.

### Updating Expectancies Based on New Social Information

We assessed how individuals who are socially anxious update their beliefs based on new social information by analyzing learning rates on the social probabilistic selection task (to measure updating expectancies about other people) and update weights on the speech expectancies task (to measure updating expectancies about one’s own social performance). There were 3 models evaluating updating: 1 for learning about other people and 2 for learning about one’s own performance (because update weights were estimated in 2 ways).

#### CBM-I’s Effects on Expectancy Updating

##### Overview

We found slightly different effects of CBM-I on expectancy updating in the 2 domains assessed. Participants in the CBM-I but not the EMA-only condition updated their expectancies about other people on the social probabilistic selection task more slowly at session 2 versus session 1. We did not find any similar main effects of CBM-I on updating expectancies about one’s social performance on the speech expectancies task. However, we found evidence of biased updating about one’s social performance in the EMA-only group on the speech expectancies task (learning more from positive than negative feedback and learning more from feedback on items related to poor vs good social performance) but did not observe these biases in the CBM-I group. This suggests that CBM-I might be associated with different changes in social RL in these 2 domains relevant to social anxiety: slower updating about other people and less-biased updating about one’s social performance (the results could also be due to other differences in the 2 tasks used besides the focus on updating about others vs oneself).

##### Slower Updating About Other People

Learning rates on the social probabilistic selection task decreased from session 1 to session 2 for participants who completed the CBM-I intervention but not for participants who completed only the EMA intervention. This suggests that CBM-I led individuals who are socially anxious to update their beliefs about other people less based on each new happy or angry face. This unexpected finding may be due to the format of the CBM-I training, in which participants read through each emotionally ambiguous scenario sentence by sentence, accumulating information slowly, and they cannot come to an emotional resolution until the final word. This intervention may work to slow learning rates by training participants to rely less on any one piece of information when figuring out what to expect of a situation or a person. Slower updating about other people is likely clinically useful in situations where the probabilities of reward and punishment are stable; another person might sometimes respond positively and sometimes negatively to different things you do, but because these probabilities would be relatively stable, it would be adaptive not to change your expectancies too much based on each new reaction from them. Individuals who are socially anxious tend to revise their impressions of others more quickly than individuals who are less anxious [57], so decreasing these learning rates, as appears to happen through CBM-I, might be beneficial. However, it is also worth considering the potential downsides of slower updating about others. If an individual who is socially anxious already has negatively biased expectancies about others (eg, “Other people will reject me.”), these biases may be corrected more slowly if the person updates their prior beliefs more slowly from new information that is more positive than expected. Although these beliefs would eventually move toward less-biased expectancies through unbiased updating from both positive and negative outcomes (ie, similar learning rates for reward and punishment, as we observed), this change would occur more slowly for people who update their beliefs about others. However, other work in this data set [37] found that CBM-I was associated with a decrease in negative interpretation bias (the target of CBM-I), suggesting that the slower updating elicited by CBM-I does not preclude a shift toward more positive beliefs in uncertain situations.

##### Less-Biased Updating About One’s Own Performance

Note that because speech expectancy updating was only measured at session 2, we cannot draw conclusions about a change in these biases as a function of CBM-I but we can consider what a difference in updating between people who completed the CBM-I intervention versus only the EMA intervention might mean. When updating expectancies of one’s own social performance based on feedback on the speech expectancies task, 2 different modeling approaches (ie, update weights estimated separately by item valence and estimated separately by prediction error valence) found that 2 different updating biases (learning more from feedback on aspects of poor vs good social performance and learning more from positive than negative feedback) occurred in the EMA-only condition but not in the CBM-I condition.

However, it is not clear that the unbiased updating observed in the CBM-I condition is uniformly beneficial, given the nature of the biases observed in the EMA-only condition, that is, the biases observed in the EMA-only condition were toward learning more from feedback on items related to poor versus good social performance and toward learning more from positive than negative feedback. The bias toward learning more from feedback on aspects of poor versus good social performance was previously found in this data set to characterize participants with high, but not low, social anxiety symptoms. This bias may be related to individuals who are socially anxious being more motivated to avoid embarrassment than to aim for excellent performance. As such, it may be good that participants who completed the CBM-I intervention did not show this bias. However, participants in the EMA-only condition also learned more from positive than negative feedback, which is likely adaptive, as it would lead to updating expectancies of one’s own social performance to be more positive, and these types of positive biases are usually associated with psychological well-being [58,59]. So, it is likely maladaptive that participants who completed CBM-I did not show this positive bias, but future work will need to determine what outcomes this and the other observed biases actually predict.

#### More Positively Biased Expectancy Updating Over Time

Because the social probabilistic selection task was administered at both sessions but the speech expectancies task was only administered at session 2, we were able to analyze changes in expectancy updating about other people over time but only had a snapshot of expectancy updating about one’s own performance at the end of the study. We found that at the end of the study, participants (regardless of intervention condition) showed more positively biased updating about other people on the social probabilistic selection task, updating their beliefs more from reward and less from punishment at session 2 than at session 1. This change over time is consistent with the session 2 snapshot of updating expectancies about one’s social performance on the speech expectancies task in the EMA-only group, who learned more from positive than negative feedback from others. These findings suggest that the EMA portion of the study, completed by all participants, might have had positive effects on social RL in both the self and other domains. Tracking one’s emotions multiple times each day for several weeks and bringing attention to one’s use of emotion regulation strategies and their associated effectiveness might have increased participants’ motivation to regulate their emotions, which might be accomplished by learning in positively biased ways. We know from prior work in this data set that trait and state social anxiety symptoms and fear of negative evaluation decreased over the course of this study, regardless of intervention condition [37], further supporting the beneficial effects of EMA about emotion regulation. However, we did not find evidence of social anxiety–linked biases in expectancy updating in prior studies using this data set (Beltzer, ML, unpublished data, June 2023) [36]. Thus, although participants with high social anxiety came to update their expectancies in more positively biased ways over the course of the study, their expectancy updating was not significantly different from that of participants with low social anxiety.

### Learning to Choose Social Reward and Avoid Social Punishment

In addition to assessing the effects of CBM-I on how individuals who are socially anxious use different types of social information to update their expectancies, we also analyzed how individuals who are socially anxious use what they have learned about other people to make decisions. This process was only measured in the domain of learning about other people and not learning about one’s own social performance. Although we did not find evidence of the hypothesized 3-way interaction, we found evidence of all the composite 2-way interactions. First, for the CBM-I condition, but not the only EMA condition, participants’ decision accuracy improved over the course of the study, such that they were able to more frequently select the more rewarding face during testing phase trials that included the most rewarding and most punishing faces. This suggests that CBM-I may improve social decision-making in individuals who are socially anxious, which may manifest in choosing to interact with people who are more likely to respond positively to them or in choosing actions that are more likely to elicit a positive response. Second, participants became better at avoiding social punishment but not at choosing social reward over the course of the study. This effect was not specific to CBM-I and suggests that either EMA about emotion regulation or practice with the task might improve accuracy in learning to avoid social punishment. This finding is particularly noteworthy because previous research, both in this data set [36] and elsewhere [17], has found that individuals who are anxious may show impairments in learning to avoid people who respond negatively to or take advantage of them. Frequent monitoring of emotions (as occurred in the EMA portion of this study) might help participants with social anxiety become more aware of when certain people make them feel bad, which might help them learn to avoid these types of punishing people.

### Clinical Implications

To the best of our knowledge, this study is the first to examine the effects of CBM-I and EMA about emotion regulation on RL. Both CBM-I and EMA are digital interventions that can be scaled up at low cost to increase access to treatment, which may help bridge the treatment gap for individuals who are socially anxious. We found that CBM-I was associated with several changes in social RL that might be helpful for individuals with social anxiety. Participants who completed CBM-I were slower to change their beliefs about others based on each instance of facial feedback. In real life, this might mean that CBM-I could help people with social anxiety resist the urge to jump to a conclusion about a person based on a single bad or good interaction. CBM-I also improved the accuracy of social decision-making about other people (as evidenced by their more frequent selection of more rewarding faces in the social probabilistic selection task at follow-up vs baseline). This higher accuracy might help individuals with social anxiety navigate social situations more successfully, resulting in more positive interactions with others and better relationships. Participants who completed the CBM-I intervention also used feedback to update their beliefs about their own social performance in less-biased ways than participants who completed only the EMA intervention, but this unbiased updating seems to be a mixed blessing. CBM-I seems to have mitigated the bias (which was observed in the EMA-only group) toward learning more from feedback on poor rather than good aspects of speech performance and having this type of unbiased updating is likely healthier for the CBM-I group (than the bias observed in the EMA-only group). However, CBM-I also seems to have mitigated the bias (that was observed in the EMA-only group) toward learning more from positive feedback than negative feedback. The EMA-only group’s bias toward learning from positive feedback would likely lead to more positive expectancies of one’s own social performance over time, which may be healthier than the CBM-I group’s unbiased learning from positive and negative feedback, although this remains to be more directly tested.

The effects observed over time regardless of the intervention condition suggest that tracking emotions and emotion regulation may also be helpful for social RL, possibly by increasing learning from positive feedback and decreasing learning from negative feedback. Given that all participants completed EMA, it is difficult to disentangle the mechanisms that might be at work. Emotion tracking might make individuals who are socially anxious more aware of the outcomes of their emotion regulation attempts, or it might encourage them to regulate in ways that promote positive affect, increasing their motivation to learn from positive social outcomes. Alternatively, they may pay more attention to when people treat them poorly and learn to make better decisions accordingly to avoid that treatment. Determining the specific mechanisms of change in social RL will be helpful in future studies.

Other studies in this data set have found that CBM-I decreased participants’ trait negative interpretation bias and increased their ability to use reappraisal day-to-day, but CBM-I was not associated with changes in cognitive flexibility and decreases in social anxiety symptoms occurred regardless of the intervention condition [37]. The fact that we found intervention-related changes in social RL and interpretation bias but nonspecific changes in symptoms raises interesting questions regarding the relationships among these cognitive, emotional, and behavioral processes. The current research design does not allow us to disentangle the temporal relations between changes in social RL and changes in interpretation bias and symptoms. However, it seems possible (and worth testing empirically in future research) that changes in social RL precede changes in social anxiety symptoms. For example, improvements in social decision-making accuracy may lead to more rewarding social interactions, which may decrease social avoidance and fear of negative evaluation.

### Limitations

There are several limitations worth noting in this study’s methodology. First, although the CBM-I intervention included mostly social scenarios, it also included several nonsocial scenarios. Its effects on social RL might have been stronger if the intervention specifically targeted negative interpretation bias in social situations. Second, this study might have been underpowered to detect the hypothesized 3-way interactions. Unfortunately, we did not conduct an a priori power analysis specific to these questions (as these data were part of a larger parent study). Relevant effect sizes in other studies with fewer participants were medium-large (eg, Cohen *d*=0.54 for participants with high social anxiety avoiding punishing faces more frequently than those with low social anxiety [13]). On the basis of a post hoc power analysis [60] using summary statistics from the study by Abraham and Hermann [13], our sample size of 106 was close to the recommended sample size of 111. However, sample sizes need to be larger to detect 3-way interactions versus the 2-way interactions tested in past studies [61]. As such, our sample may have been appropriately powered to detect 2-way interactions but may have been underpowered to detect higher-degree interactions. Third, some characteristics of our sample, including their relative homogeneity in age, race, and ethnicity and that they were not clinically diagnosed with social anxiety disorder, limit generalizability to clinical populations more broadly. Fourth, because we were only able to ethically collect data on social RL about one’s own social performance at the end of the study, we do not know if there were baseline differences in performance on this task between the CBM-I and EMA-only groups.

### Future Directions

Given our initial findings of several positive changes in social RL associated with CBM-I, future research may help illuminate the mechanisms of change as well as how changes in social RL relate to changes in other important constructs, such as reduced anxious avoidance. Furthermore, studies may look at how social RL changes over the course of interventions other than CBM-I, such as cognitive behavioral or interpersonal therapy, may predict response to treatment. Future studies may also incorporate passive sensing to determine the optimal time and context to deliver CBM-I as a just-in-time adaptive intervention, reaching people when it can help them the most [62]. Updating rigidly negative expectancies and beliefs to effectively consider a range of feedback and information is key to healthy interpersonal and emotional functioning; continued research on social RL in social anxiety may help us understand why certain biases persist and how we might work to change them.

## Appendix

Multimedia Appendix 1 CONSORT (Consolidated Standards of Reporting Trials) diagram.

Multimedia Appendix 2 Screenshots of cognitive bias modification for interpretations training.

Multimedia Appendix 3 Supplementary methods, results, tables, and figures.

## Abbreviations

CBM-I: cognitive bias modification for interpretations
CONSORT: Consolidated Standards of Reporting Trials
EMA: ecological momentary assessment
LOOIC: leave-one-out information criterion
RL: reinforcement learning

## Data Availability

The data sets analyzed during this study are available on the web [38].
