# Supplementary material for: Changes in Learning From Social Feedback After Web-Based Interpretation Bias Modification: Secondary Analysis of a Digital Mental Health Intervention Among Individuals With High Social Anxiety Symptoms
Source: JMIR Form Res. 2023 Aug 9;7:e44888. doi: 10.2196/44888 (PMC10448289; doi:10.2196/44888)
Supplement: Multimedia Appendix 1 [file formative_v7i1e44888_app1.docx]

**Multimedia Appendix 1: CONSORT Diagram**

Returned for Session 2 lab follow-up (n=49), including social probabilistic selection task and speech expectancies task

- Failed to show up for Session 2 (n=2)

Returned for Session 2 lab follow-up (n=50), including social probabilistic selection task and speech expectancies task

- Dropped out in Week 1 of EMA (n=2)
- Dropped out in Week 3 of EMA (n=2)
- Failed to show up for Session 2 (n=1)

Completed Session 1 in lab, including social probabilistic selection task and speech expectancies task

(Note, n=43 low SA also completed the lab session, and their data were included in Studies 1 and 2, but the low SA group is not part of Study 3.)

Allocated to EMA-only (n=55 high SA)

Allocated to CBM-I (n=59 high SA)

Randomized (n=114 high SA)

Did not initiate CBM-I (n=8)

- Dropped out prior to intervention (n=3)
- Declined to initiate intervention (n=5)

Initiated CBM-I (n=51)

Engaged in EMA-only (n=55)

Excluded (n=1,730)

- Not meeting inclusion criteria (n=964)
- Qualifying online survey responders who declined to participate (n=3)
- Qualifying online survey responders that were not contacted (n=100)
- Qualifying participant pool registrants that did not sign up (n=663)

Assessed for eligibility (n=1,844)

- Online survey responders (n=364)
- University participant pool registrants (n=1,480)
