# Supplementary figures and images for: Changes in Learning From Social Feedback After Web-Based Interpretation Bias Modification: Secondary Analysis of a Digital Mental Health Intervention Among Individuals With High Social Anxiety Symptoms
Source: JMIR Form Res. 2023 Aug 9;7:e44888. doi: 10.2196/44888 (PMC10448289; doi:10.2196/44888)

**Multimedia Appendix 2:Screenshots of CBM-I**


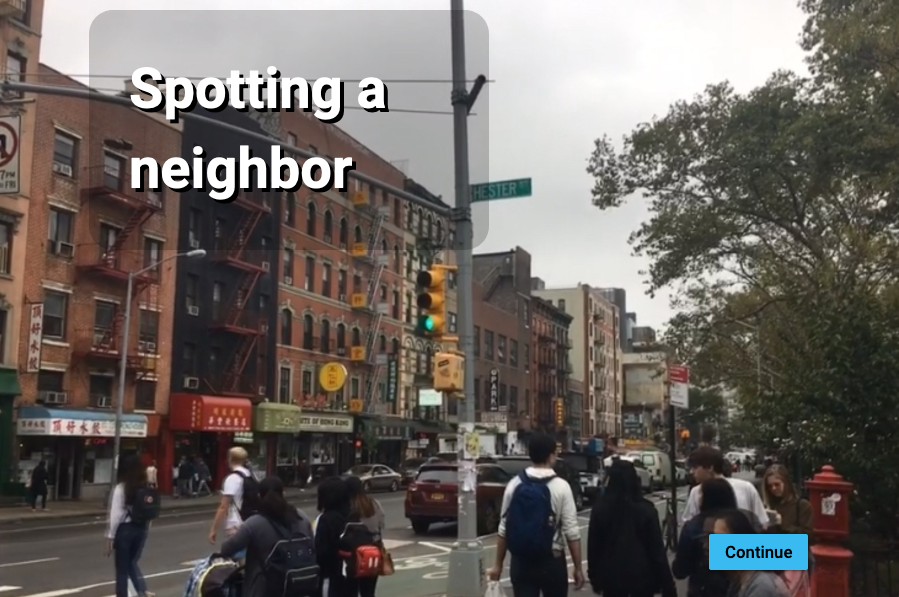


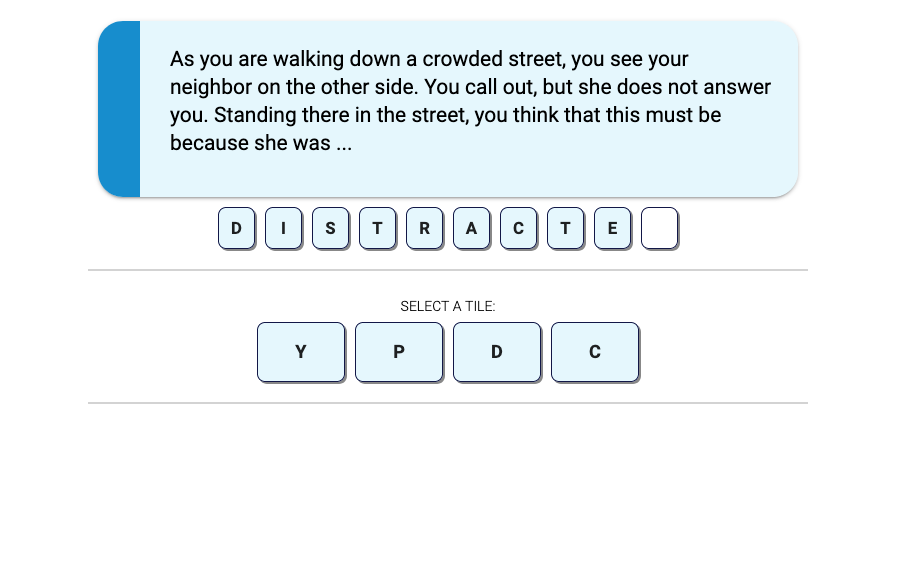


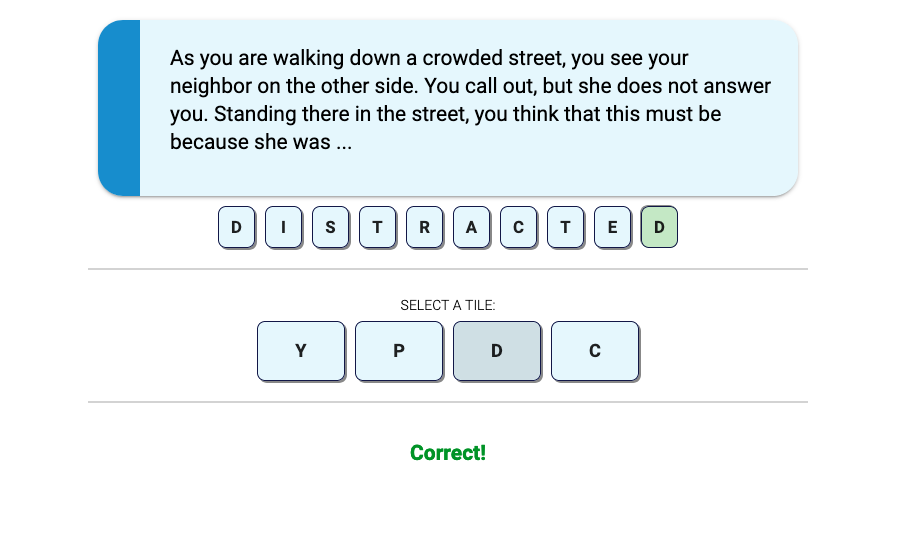


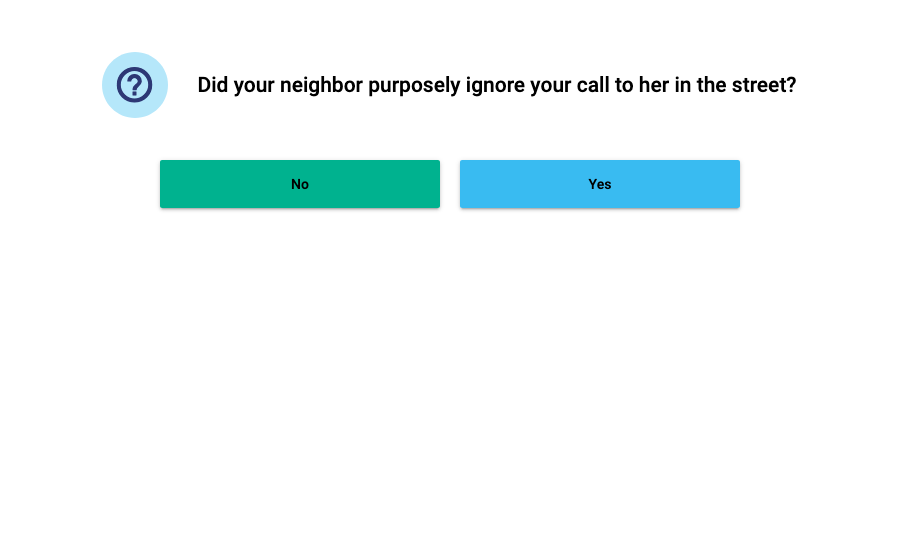

Supplement: Multimedia Appendix 2 [file formative_v7i1e44888_app2.docx]
