# Supplementary material for: Changes in Learning From Social Feedback After Web-Based Interpretation Bias Modification: Secondary Analysis of a Digital Mental Health Intervention Among Individuals With High Social Anxiety Symptoms
Source: JMIR Form Res. 2023 Aug 9;7:e44888. doi: 10.2196/44888 (PMC10448289; doi:10.2196/44888)
Supplement: Multimedia Appendix 3 [file formative_v7i1e44888_app3.docx]

**Multimedia Appendix 3: Supplementary Method, Results, Tables, and Figures**

**Supplementary Method**

**Recruitment**

Recruitment flyers and emails did not mention the CBM-I intervention or describe the study as a treatment study. In the consent form, CBM-I was described as: “Some participants will be assigned to complete six 10-minute/day online activities during the third week that involve reading sentences and filling in missing letters from word fragments. (Time: approximately 1 hour). This will be determined by random assignment, so there is equal chance to be in either condition. If you are assigned to the condition that does not receive the training program and you would like to try the program, you will be offered the program for free at the end of your study participation.”

**Measures**

***Social Probabilistic Selection Task***

The training phase consisted of two to six blocks of 60 trials each, with 20 trials of each pair of faces in each block (10 with the more rewarding face on each side). Participants advanced to the testing phase either once they had reached the maximum number of training blocks or they had met performance criteria, with higher accuracy required on more difficult face pairs (based on prior work [44]). The testing phase consisted of 120 trials, with face pair shown eight times (four with the more rewarding face on each side).

Two sets of face images [45,46] were used as stimuli so participants saw different faces at the two sessions, with their order counterbalanced. White faces of men were shown to female participants and White faces of women were shown to male participants based on research finding effects of facial stimuli sex [13,47] and race [48] on social RL. Faces whose expressions of happiness, anger, and neutral emotion were well identified by previous samples [45,46] were selected for inclusion as stimuli. For each participant, the faces were randomized to the six reward/punishment contingencies. The SPST was presented in MATLAB.

***Speech Expectancies Task***

A funnel debriefing was used to check for suspicion about the false feedback. Participants answered open-ended questions that started with more general feedback about the study and ended with the most specific question, “Did you believe the judges’ feedback on your speech?”

**Plan for Analyses**

***Computational Modeling of the Social Probabilistic Selection Task***

Two candidate Q-learning models were compared, one with separate learning rate parameters for rewarding and punishing feedback, and one with a single learning rate parameter. In the more complex model, each prediction error was multiplied by a learning rate $\alpha$ to update *Q* values following:

$$Q_{i}\left( t+1 \right)=Q_{i}\left( t \right) +{\alpha_{R}\left[ r\left( t \right)- Q_{i}\left( t \right) \right]}_{+}+ \alpha_{P}\left[ r\left( t \right)- Q_{i}\left( t \right) \right]_{-}$$

Where *r(t) =* 1 for rewarding (happy face) and 0 for punishing (angry face) feedback. Positive prediction errors occurred when the outcome was better than expected, and the learning rate $\alpha_{R}$ was applied. Negative prediction errors occurred when the outcome was worse than expected, and the punishment learning rate $\alpha_{P}$ was applied.

The simpler model updated *Q* values with a single learning rate$\alpha$ for both positive and negative prediction errors following:

$$Q_{i}\left( t+1 \right)=Q_{i}\left( t \right) + \alpha\left[ r\left( t \right)- Q_{i}\left( t \right) \right]$$

To determine the probability of a participant selecting a given face in each pair, *Q* values were entered into the following softmax equation with inverse temperature $\beta$:

$$P_{A}(t)=\frac{e^{{Q_{A}}/\beta}}{e^{{Q_{A}}/\beta}+e^{{Q_{B}}/\beta}}$$

We ensured that MCMC chains were well mixed and converged to stationary distributions for stable parameter estimates, checked whether $\hat{R}$ values for parameters were approximately 1, produced and examined trace plots of the group-level parameters, and plotted and visually examined posterior distributions of the group- and individual-level parameters.

***Computational Modeling of the Speech Expectancies Task***

Each update weight *b* was estimated according to:

post-rating ~ pre-rating + *b*(feedback - pre-rating)

where pre-rating is the participant’s rating of how they expected to perform on an item before giving a speech, feedback is the false judges’ rating that they were shown on that item after their speech, and post-rating is the participant’s rating for how they expected they would perform on that item during a similar speech in the future. Our two main models estimated two separate update weights *b;* in one model, there were separate update weights for items assessing good versus poor speech performance, and in the other model, there were separate update weights for positive (feedback - pre-rating > 0) versus negative prediction errors (feedback - pre-rating < 0). Additional models estimated four update weights (one for each combination of item and prediction error valence) and one update weight across all 20 trials. The results of these additional models are reported in this Supplement.

**Supplemental Results**

**Data Reduction**

Of the 59 participants assigned to the CBM-I group, three dropped out of the study prior to CBM-I and five declined to initiate CBM-I but remained in the study; these participants were removed from analyses. This left 51 participants who completed at least one CBM-I session (and *n*=55 in the EMA-only group), who were included in analyses regardless of how many additional CBM-I sessions they completed. Of these, 41 completed all six CBM-I training sessions, two completed five sessions, one completed four sessions, three completed three sessions, two completed two sessions, and two completed one session.

Of the remaining 106 participants who completed the social probabilistic selection task once, data were missing at the other point for 10 participants, which meant that we could not use them for analyses. Four participants dropped out of the study between sessions (all EMA-only), three did not attend the second session (two CBM-I, one EMA-only), and four participants’ data was lost due to technical problems (e.g., computer freezing) or experimenter error. This resulted in a final sample of *N=*96 (*n=*47 in the CBM-I group and *n*=49 in the EMA-only group) for the social probabilistic selection task. For the speech expectancies task, three participants declined to complete the speech at the second session. Of those with complete speech expectancies task data, 18 participants indicated during the funnel debriefing that they did not believe the judges’ feedback and were thus excluded from analyses, resulting in a final sample of *N=*78 (*n*=36 in the CBM-I group and *n*=42 in the EMA-only group) participants included in analyses for the speech expectancies task.

**Analyses of the Social Probabilistic Selection Task (RL About Other People)**

***Accuracy Measured During the Testing Phase (Interaction Effect That Does Not Include Session Variable)***

Following up on the significant condition-by-trial type interaction, we found that accuracy in choosing reward was higher than accuracy in avoiding punishment for both the CBM-I (choose reward: *M* = 1.93, *SD* = 0.13; avoid punishment: *M* = 1.20, *SD* = 0.13, *OR* = 10.84, *p* < 0.001, *d* = 0.73) and EMA-only conditions (choose reward: *M* = 1.66, *SD* = 0.12; avoid punishment: *M* = 1.26, *SD* = 0.12, *OR* = 6.37, *p* < .001, *d* = 0.40; see Figure S1). In other words, regardless of session, participants in both conditions made better decisions in the testing phase when selecting from a pair that included the most rewarding face instead of the most punishing face. Notably, examination of the effect sizes and visual inspection of the interaction plots suggested that this difference between accuracies in choosing reward and avoiding punishment was greater in the CBM-I (vs. EMA-only) condition. This result is partially in line with hypotheses based on prior literature. We hypothesized that learning biases would become less negative or more positive with CBM-I, and we found that learning biases were more positive in the CBM-I condition versus EMA-only (but regardless of session).

***Exploratory Bayesian Comparisons***

Given the Bayesian computational modeling approach, group-level posterior distributions, and not just individual-level point estimates, were estimated for learning rates. Exploratory pairwise comparisons were performed on these posterior distributions to better understand possible learning rate differences in this sample by computing the 95% highest density interval (HDI) of the difference between the two posterior distributions.

Comparing learning rates across sessions within the EMA-only condition, the HDI for reward learning rates did not cross zero [0.0075, 0.2025], but the HDI for punishment learning rates did [-0.0709, 0.1317], suggesting that reward, but not punishment, learning rates credibly increased from session one to session two. Comparing across sessions within the CBM-I condition, HDIs for both reward [-0.0021, 0.0187] and punishment learning rates [-0.1732, 0.019] both crossed zero, but just barely. This suggests, for the CBM-I condition, that there was a trend towards reward learning rates increasing and punishment learning rates decreasing from session one to session two, but that it was not quite at 95% credibility.

Comparing learning rates across prediction error valence at each session within the EMA-only condition, the HDIs at session one [-0.1455, 0.0705] and session two [-0.0623, 0.1405] both crossed zero, suggesting no credible difference between reward and punishment learning rates at either session. Comparing across prediction error valence at each session within the CBM-I condition, the HDI at session one [-0.1851, 0.0378] crossed zero, though just barely, but the HDI at session two did not [0.0148, 0.1787]. This suggests that, for the CBM-I group, at session one, there was a trend towards reward learning rates being lower than punishment learning rates, but at session two, reward learning rates became credibly higher than punishment learning rates. Taken together, these exploratory results suggest a pattern (though not all at 95% credibility) of higher punishment than reward learning rates at baseline, with reward learning rates increasing between sessions for both conditions but punishment learning rates increasing only for the CBM-I condition, such that reward learning rates were credibly higher than punishment at session two for those in the CBM-I condition.

**Analyses of the Speech Expectancies Task (RL About One’s Own Social Performance)**

***Update Weights Estimated Separately by Prediction Error and Item Valence***

To compare expectancy updating between social anxiety groups, a linear mixed effects model was performed predicting update weights, with update weights estimated separately over positive and negative prediction errors and items measuring good versus poor social performance (so, estimating four update weights per participant). This model included fixed effects of CBM-I condition, item valence, and prediction error valence, and all two- and three-way interactions, with a random intercept for participant. Four observations (1.5% of the data) were identified as influential outliers (classified as those whose Cook’s distance was greater than four divided by the number of observations), and removed [49]. The model’s fit was singular, so the random intercept was dropped and a linear model was tested. The only statistically significant effect was a main effect of prediction error valence, such that participants more heavily weighted positive (vs. negative) feedback when updating their expectancies about their social performance (Figure S2). See Table S2.

***Update Weights Estimated Over All Items Together***

A t-test was performed comparing a single update weight estimated over all items between CBM-I conditions, and found no significant difference between the EMA-only (*M* = 0.45) and CBM-I (*M* = 0.53) conditions (*t*(74.25) = -1.33, *p* = .19, *d* = 0.30). When speech expectancy updating was modeled with no regard for item or prediction error valence, no differences were found between intervention groups.

**Table S1**

***LOOIC of Candidate Models***

|  | EMA-only, S1 | EMA-only, S2 | CBM-I, S1 | CBM-I, S2 |
| --- | --- | --- | --- | --- |
| Two LRs | 12352.45 | 10071.98 | 10367.72 | 7390.60 |
| Single LR | 12398.83 | 10072.02 | 10358.86 | 7385.28 |

**Table S2**

*Model Estimates Predicting Update Weights Estimated* *Separately by Prediction Error and Item Valence in the Speech Expectancies Task*

|  | **Update Weight** | | | |
| --- | --- | --- | --- | --- |
| *Predictors* | *Estimates* | *CI* | *Statistic* | *p* |
| (Intercept) | 0.56 | 0.45 – 0.66 | 10.63 | **<0.001** |
| Condition | 0.03 | -0.08 – 0.13 | 0.50 | 0.617 |
| PE Valence | 0.10 | 0.00 – 0.21 | 2.00 | **0.047** |
| Item Valence | -0.04 | -0.14 – 0.07 | -0.69 | 0.492 |
| Condition X PE Valence | 0.05 | -0.05 – 0.16 | 1.00 | 0.318 |
| Condition X Item Valence | -0.00 | -0.11 – 0.10 | -0.09 | 0.928 |
| PE Valence X Item Valence | 0.05 | -0.05 – 0.15 | 0.96 | 0.340 |
| Condition X PE Valence X X Item Valence | -0.06 | -0.16 – 0.05 | -1.11 | 0.268 |
| Observations | 281 | | | |
| R^2^ / R^2^ adjusted | 0.027 / 0.002 | | | |

Note: Condition = intervention condition (CBM-I vs. EMA-only). PE Valence = prediction error valence (positive vs. negative). Item Valence = items measuring good vs. poor social performance.

**Figure S1**

**
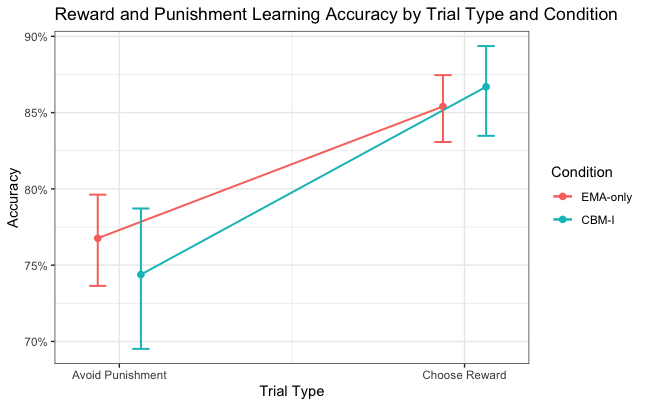
**

Note: Condition = intervention condition (CBM-I vs. EMA-only).

**Figure S2**

***Update Weights in the 4-Weight Model for the Speech Expectancies Task***

**
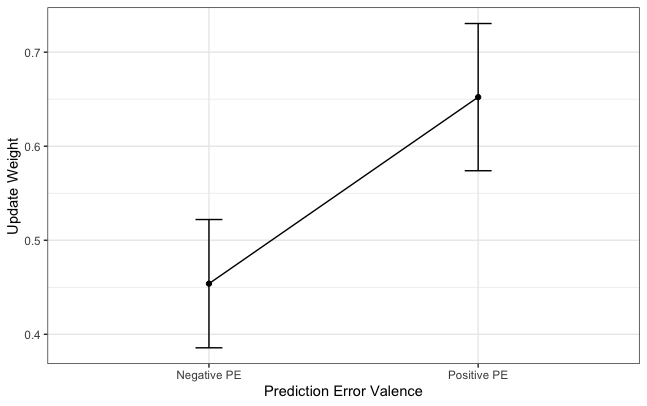
**

Note: In the 4-weight model, update weights were estimated separately for items measuring good vs. poor social performance on which feedback was more negative vs. positive than participants’ pre-speech expectancies (prediction error valence).
